# *Notes from the Field:* Characteristics of Tetrahydrocannabinol–Containing E-cigarette, or Vaping, Products Used by Adults — Illinois, September–October 2019

**DOI:** 10.15585/mmwr.mm6929a5

**Published:** 2020-07-24

**Authors:** Livia Navon, Isaac Ghinai, Jennifer Layden

**Affiliations:** ^1^Illinois Department of Public Health; ^2^Center for Preparedness and Response, CDC; ^3^Epidemic Intelligence Service, CDC; ^4^Chicago Department of Public Health, Illinois.

As of February 18, 2020, 2,807 patients hospitalized with e-cigarette, or vaping, product use–associated lung injury (EVALI) had been reported to CDC (*1*). Nationwide, and in Illinois, approximately 80% of EVALI patients reported use of tetrahydrocannabinol (THC)-containing e-cigarette, or vaping, products (*2*,*3*). The recent EVALI outbreak highlighted the limited availability of data on the characteristics of THC-containing e-cigarette, or vaping, products used in the United States.

During the EVALI outbreak, the Illinois Department of Public Health (IDPH) developed an online public survey targeting Illinois adults aged ≥18 years who used any e-cigarette, or vaping, products (*4*). The survey included questions about e-cigarette, or vaping, product use in the past 3 months, including types of substances used (e.g., nicotine, THC), product brand names (respondents could list up to 10 products), types of devices used (e.g., tank models, dab rigs), and product forms (e.g., oils, solids). The public survey link was available on the IDPH website during September 17–October 8, 2019, and was publicized by IDPH, the news media, and local health departments.

Overall, 4,527 survey responses were received from residents of all 102 Illinois counties; 939 (21%) respondents reported use of THC-containing e-cigarette, or vaping, products during the past 3 months; the median age of these respondents was 34 years (range = 18–77 years). Among THC-containing product users, 501 (53%) provided the brand names of products they had used in the past 3 months. These 501 respondents reported using 732 THC-containing products with 220 different brand names. Fifty-eight brands (26%) were reported by more than one respondent and accounted for 78% (570 of 732) of products reported, with the remaining 162 brand names each reported by only one respondent. Dank Vapes, a class of illicit THC-containing products sold under the same brand name but with no obvious centralized production or distribution, was the most commonly reported brand name (151 of 732 products; 21%) followed by Cresco* (59 of 732; 8%) (Figure). Products available through the Illinois Medical Cannabis Patient Program accounted for 23% of reported products (169 of 732 products); survey respondents aged ≥35 years reported 63% (106 of 169) of these legally available products.

**FIGURE:**
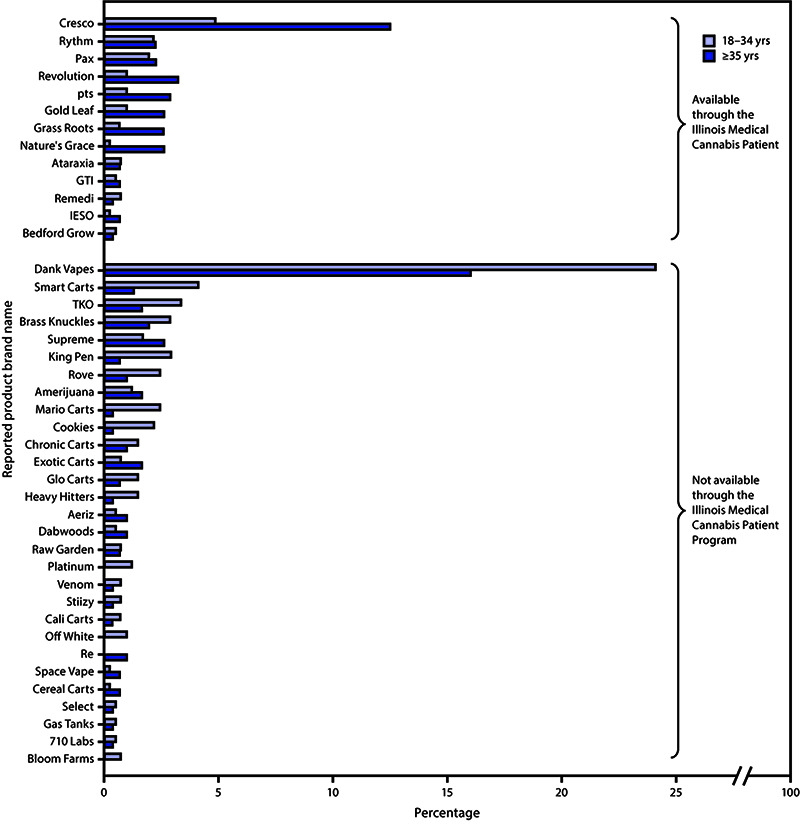
Most frequently reported tetrahydrocannabinol (THC)-containing e-cigarette, or vaping, product brand names* as a percentage of all named products, by age group^†^ and by Illinois Medical Cannabis Patient Program availability^§^ among a convenience sample of adult e-cigarette, or vaping, product users — Illinois, September–October 2019 * Brand names reported by at least three survey respondents are displayed. ^†^ Survey respondents aged 18–34 years reported 419 products with brand names; survey respondents aged ≥35 years reported 313 products with brand names. Percentages for each age group were calculated using these denominators. ^§^ A full list of products licensed through the Illinois Medical Cannabis Patient Program is available at https://www2.illinois.gov/sites/agr/Plants/MCPP/Pages/default.aspx.At the time of the survey, products not available through the Illinois Medical Cannabis Patient Program were likely obtained through informal sources such as friends, family, in-person or online dealers, or from in-person purchases in jurisdictions with legalized adult-use cannabis sales. In Illinois, legal sale of adult-use cannabis products from licensed dispensaries began on January 1, 2020.

Overall, 638 (68%) THC-containing product users reported which product form they used. Among these 638 respondents, 501 (79%) reported using prefilled, oil-containing cartridges, and 47 (7%) reported using THC-containing oil not in prefilled cartridges. Use of solids, such as dabs or waxes, was reported by 124 (19%) respondents, and use of marijuana plant material in e-cigarette, or vaping, devices was reported by 21 (3%) respondents. Fourteen percent of respondents (92 of 638) reported using more than one product form. Among the 695 THC-containing product users who provided e-cigarette, or vaping, device information, 244 (35%) reported using more than one type of device.

Although these data are from a convenience sample, these findings highlight the diversity of available THC-containing e-cigarette, or vaping, products. Most consumers of these products reported using prefilled, oil-containing cartridges; however, use of multiple product forms and device types was reported. Product brands used likely vary across jurisdictions and the corresponding regulatory environments for THC-containing products. Dank Vapes, the brand most frequently reported by survey respondents, was also the brand most frequently reported by EVALI patients in Illinois and nationally (*2*,*3*). To reduce the risk of EVALI, people should not use THC-containing e-cigarette, or vaping, products, particularly from informal sources such as friends, family, or in-person or online dealers (*1*).

## Footnotes

* Cresco is a product brand available legally through the Illinois Medical Cannabis Patient Program. A full list of products licensed through the Program is available at: https://www2.illinois.gov/sites/agr/Plants/MCPP/Pages/default.aspx.
